# Combining Soil Databases for Topsoil Organic Carbon Mapping in Europe

**DOI:** 10.1371/journal.pone.0152098

**Published:** 2016-03-24

**Authors:** Ece Aksoy, Yusuf Yigini, Luca Montanarella

**Affiliations:** 1 European Topic Center-Urban Land Soil, University of Malaga, Malaga, Spain; 2 Institue for Environment and Sustainability, Joint Research Center of European Commission, Ispra, Italy; Chinese Academy of Forestry, CHINA

## Abstract

Accuracy in assessing the distribution of soil organic carbon (SOC) is an important issue because of playing key roles in the functions of both natural ecosystems and agricultural systems. There are several studies in the literature with the aim of finding the best method to assess and map the distribution of SOC content for Europe. Therefore this study aims searching for another aspect of this issue by looking to the performances of using aggregated soil samples coming from different studies and land-uses. The total number of the soil samples in this study was 23,835 and they’re collected from the “Land Use/Cover Area frame Statistical Survey” (LUCAS) Project (samples from agricultural soil), BioSoil Project (samples from forest soil), and “Soil Transformations in European Catchments” (SoilTrEC) Project (samples from local soil data coming from six different critical zone observatories (CZOs) in Europe). Moreover, 15 spatial indicators (slope, aspect, elevation, compound topographic index (CTI), CORINE land-cover classification, parent material, texture, world reference base (WRB) soil classification, geological formations, annual average temperature, min-max temperature, total precipitation and average precipitation (for years 1960–1990 and 2000–2010)) were used as auxiliary variables in this prediction. One of the most popular geostatistical techniques, Regression-Kriging (RK), was applied to build the model and assess the distribution of SOC. This study showed that, even though RK method was appropriate for successful SOC mapping, using combined databases was not helpful to increase the statistical significance of the method results for assessing the SOC distribution. According to our results; SOC variation was mainly affected by elevation, slope, CTI, average temperature, average and total precipitation, texture, WRB and CORINE variables for Europe scale in our model. Moreover, the highest average SOC contents were found in the wetland areas; agricultural areas have much lower soil organic carbon content than forest and semi natural areas; Ireland, Sweden and Finland has the highest SOC, on the contrary, Portugal, Poland, Hungary, Spain, Italy have the lowest values with the average 3%.

## Introduction

Numerous environmental and socio-economic models require soil parameters as inputs to estimate and forecast changes in our future life conditions. However, the availability of soil data is limited on both national and European scales. Soil information is either missing at the appropriate scale, its meaning is not well explained for reliable interpretation, or the quality of the data is questionable [1]. There are several reasons for working on assessing the distribution of this important chemical parameter, such as; SOC is a quantifiable indicator which is of high importance for evaluating the state of soils in Europe; SOC is of high interest for environmental policy making in Europe; existence of comparable modelling datasets exist at local/national and European level; and availability of auxiliary datasets (environmental covariates) for the best application of a modelling platform.

Digital soil mapping (DSM) has evolved as a discipline linking field, laboratory, and proximal soil observations with quantitative methods to infer on spatial patterns of soils across various spatial and temporal scales. Studies use various approaches to predict soil properties or classes including univariate and multi-variate statistical, geostatistical and hybrid methods, and process-based models that relate soils to environmental covariates considering spatial and temporal dimensions [2].

Geostatistical techniques allow for the prediction of soil properties using soil information and environmental covariates. There are some commonly used geostatistical methods (Inverse Distance Weighted (IDW), Multiple Linear Regression, Ordinary Kirging, Co-Kriging, Radial Basis Functions (RBF), Geographical Weighted Regression, Partial Least Squares Regression, Regression Kriging (RK), etc.) to map soil properties in the literature. RK method as one of the widely used geostatistical techniques has been used for producing of soil property maps [3–12]. RK is a spatial interpolation technique that combines a regression of the dependent variable on auxiliary variables (such as land surface parameters, remote sensing imagery and thematic maps) with simple kriging of the regression residuals. In other words, RK is a hybrid method that combines either a simple or a multiple-linear regression model with ordinary, or simple, kriging of the regression residuals [13, 6]. RK is becoming an important tool in geostatistics because of its user-friendliness and its accuracy often outperforms ordinary linear regression and ordinary kriging [8].

The European Commission is currently funding a $10-million CZO programme with 10 sites in Europe, the United States and China focused on mitigating soil threats. Four core European sites represent key stages of the soil cycle. At the Damma Glacier CZO in Switzerland, researchers are studying the stages of development of new soil formed over the past 150 years on bedrock exposed as the glacier retreats due to global warming. The Fuchsenbigl CZO in Austria is dedicated to studying the development of soil fertility on a floodplain: sediments deposited along the Danube River since the last glaciation reveal progressive stages of soil formation over thousands of years. The Lysina CZO in the Czech Republic is focused on soil recovery in managed forests, in an area damaged by acid rain during the late twentieth century. The Koiliaris CZO in Crete, Greece, has mature soils affected by millennia of agriculture and is under imminent threat of desertification because of global warming [14]. The aim of this study was aggregating those different databases which were coming from local (CZOs) and EU scale databases (LUCAS and Biosoil) and comparing the effects of different combinations of the datasets and searching for how the performances might change.

## Material and Method

### Materials

**Fig 1:**
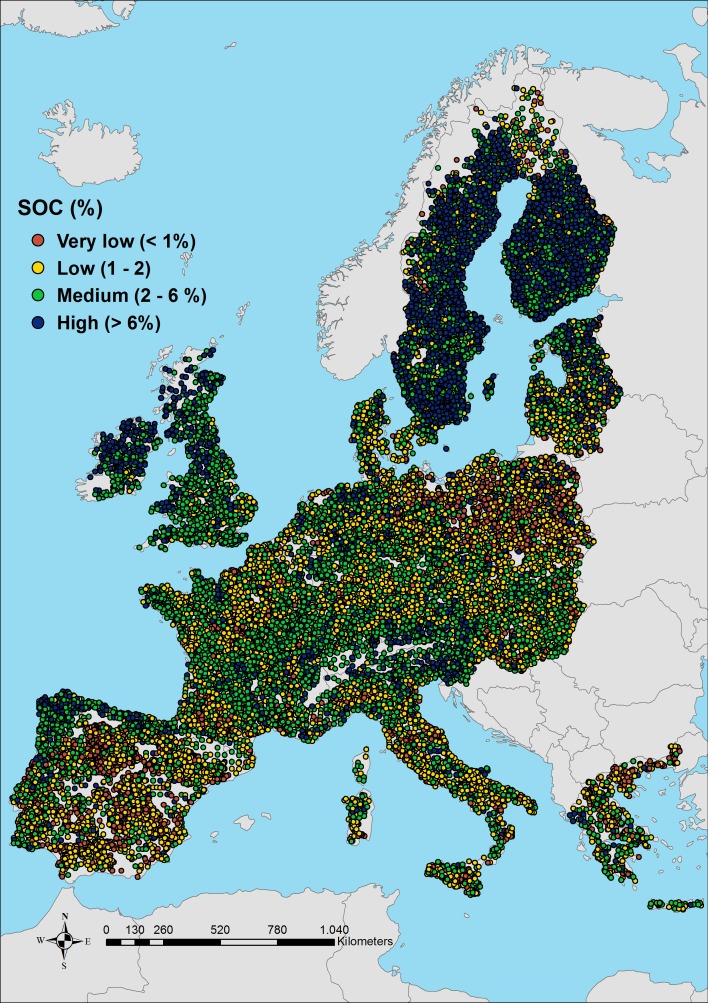
Distribution of SOC samples. Very low (<1%) Low (1–2%) Medium (2–6%) High (> 6%).

The main dataset used in this study is made up of totally 23,835 soil samples collected from three different studies on different land-uses: 19,860 points from the LUCAS Project (samples from agricultural soil); 3588 points from the Biosoil Project (samples from forest soil); and 387 samples from the SoilTrEC Project (samples from local soil data coming from six different critical zone observatories (CZOs) in Europe). The distribution of the soil organic carbon measurements can be seen Fig 1.

### Soil Samples

#### LUCAS dataset

Land Use/Cover Area frame Statistical Survey (LUCAS) [15, 16] is an in-situ survey, which means that the data are gathered through direct field observations. The aim of the LUCAS survey is to gather fully harmonized data on land use/cover and their changes over time in the EU 27. In the LUCAS (2009) survey, 265,000 geo-referenced points were visited by more than 500 field surveyors. The survey points were selected from a standard 2 km × 2 km grid based on stratification information provided by Martino&Fritz [17].

For the first time the LUCAS (2009) survey included a soil module. Top soil samples (0–30 cm) were collected from 10% of the survey points, thus providing approximately 20,000 soil samples. LUCAS soil samples were taken from all land use/land cover types; however, the survey focused mainly on agricultural areas. Each soil sample was taken from the topsoil zone (top 30 cm) with a weight of ca. 0.5 kg. The objective of the soil module was to improve the availability of harmonized data on soil parameters in Europe. The 19,860 LUCAS soil samples were analysed in a single ISO-certified laboratory that used harmonized chemical and physical analytical methods (ISO standards, or their equivalent) in order to obtain a coherent and harmonized dataset with pan-European coverage. The analysis results formed the LUCAS soil database, including, inter alia, SOC in top soils (0–30 cm) expressed in g/kg [18]. For the determination of the organic carbon content correction for LUCAS soil samples is made with the carbonate content determined according to ISO 10694:1995 [19].

#### Biosoil Dataset

It was a research challenge for the project to use additional data covering the forest land use which was not sampled adequately in LUCAS survey. For this purpose, JRC has taken into account the Biosoil study carried out within the scope of the Forest Focus EC regulation 2152⁄2003 under the responsibility of the Institute for Environment and Sustainability of the European Commission Joint Research Centre. The aim of the project was to demonstrate the feasibility of harmonized monitoring of forest soils at the European scale involving 22 countries and following common manuals [20]. As the project monitored forest and environmental interactions at European level [21, 22], the Soil Organic carbon samples were taken during the Biosoil Survey in 2006. The result of Biosoil project was the Biosoil dataset with 3,379 plots across Europe.

#### SoilTrEC Organic Carbon Dataset

**Fig 2:**
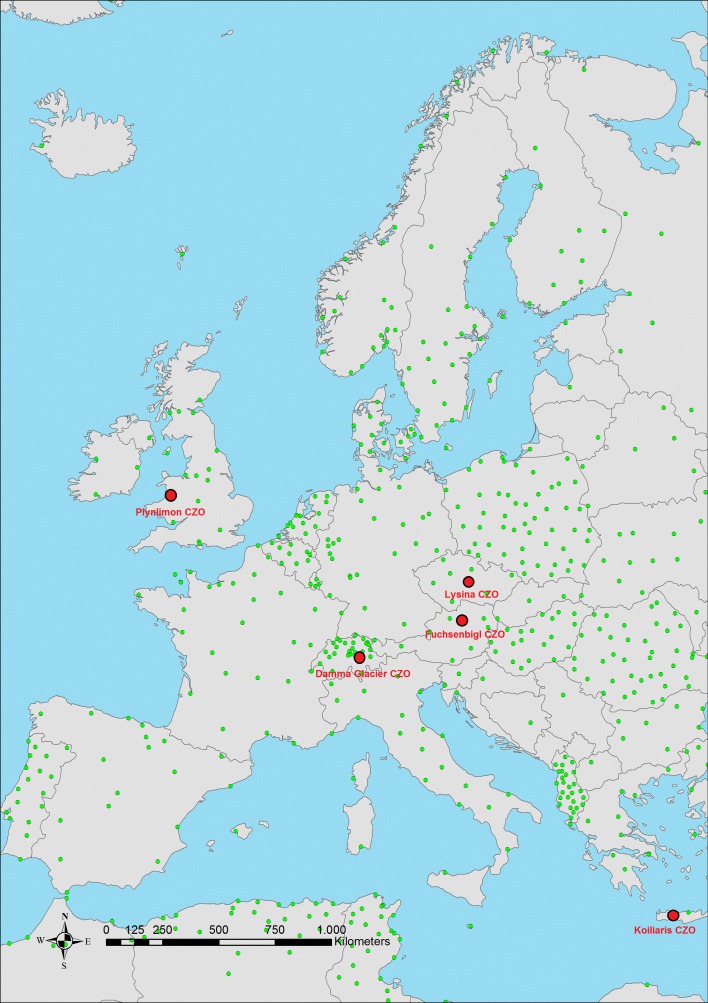
Locations of CZOs in SoilTrEC Project CZOs.

SoilTrEC Dataset is originated from the Critical Zone Observatories (CZOs) in SoilTrEC Project. SOC measurements from five different located CZOs in Europe (Fig 2) (60 samples from Koiliaris (Greece) [23], 33 samples from Damma Glacier (Switzerland) [24], 33 samples from Lysina (Czech Republic) [25], 71 samples from Fuchsenbigl (Austria) [26], 85 samples from Plynlimon (UK)) are merged and included in this study. Besides CZOs data, 105 samples from Switzerland (data were measured between 2000 and 2004 in the fourth repeated sampling campaign of Swiss Soil Monitoring Network (NABO) [27] are used to fill the gap between geographical borders of Europe Content.

### Auxiliary Variables

Different variables can be used for different study areas to best explanation of SOC distribution. Either one or all of the factors together might be found as significant and might have changed SOC content. The combination and the correlation of the significant variables which effect SOC content might be different in the different regions.

Generally, climate (temperature, precipitation, topographic/compound wetness index, evaporation, soil moisture, etc.), topography (slope, aspect, elevation, etc.), soil texture, parent material, geology, vegetation (NDVI, etc.) and land use types are used as environmental covariates for predicting soil organic carbon content. Both continuous (slope, aspect, temperature, precipitation) as well as categorical (elevation, geology, land-cover map, soil map) factors were used as auxiliary variables in our study to predict distribution of SOC and to map it as a spatially continuous surface as listed below:

1. DEM (90 m resolution, SRTM)
  1.1 Slope (%)
  1.2 Aspect
  1.3 Elevation
  1.4 CTI (Compound topographic index)
2. Soil map (European Soil Bureau Network (ESBN) Database [28], JRC, WRB-Level1 Classes)
3. Geology Map (IGME 5000. 1/5Million International Geological Map of Europe and Adjacent Areas)
4. Land-Cover Map (CORINE 2000, EEA) [29]
5. Soil texture map (ESBN Database, JRC, Texture Classes) [28]
6. Parent material map (ESBN Database, JRC, dominant parent material classes) [28]
7. Climatic Data
  7.1 WorldClim Data (EFSA & JRC, 1km) [30–32, 18]
    7.1.1 Total precipitation (1950–2000 years, total precipitation)
    7.1.2 Average precipitation (1960–1990 years, average monthly precipitation, mm/month)
    7.1.3 Average temperature (1960–1990 years, average monthly mean temperature)
  7.2 AGRI4CAST Interpolated Meteorological data (MARS Unit, JRC, 25km)
  7.2.1 Average precipitation (2000–2010 years, Average total precipitation)
  7.2.2 Max temperature (2000–2010 years, Average annual max temperatures)
  7.2.3 Min temperature (2000–2010 years, Average annual min temperatures)

### Data preparation and processing

All input data were being prepared before executing geostatistical analysis; all input data were prepared using transformations for compliant projection and coordinate system (ETRS_1989_LAEA) and were resampled to the same resolution (90m). These actions ensured compatible data structure.

All covariates were normalized before executing the model.Most of the continuous covariates (slope, temperature, precipitation, etc.) was normalized by using Z-score normalization technique. The range of [-1, 1] was used for aspect instead of [0, 360], by taking their sinus. The number of classes–were kept between 7 and 8- in order to reduce the categorical information as well as the importance of a specific covariate. Expert knowledge was used in this process. Categorical covariates were normalized by reclassifying the chosen main classes and then transferring these classes into new layers. The reclassification process resulted in binary data (0 and 1 classes) for each layer.

Fifteen different variables were used to assess and model the relationship between SOC and environmental factors as given in auxiliary variables section. The detail description of the preparation processes for each of the variables can be found in further paragraphs in this section.

Topographic covariates were obtained from a DEM which comes from SRTM 100m digital terrain model: elevation, slope gradient (%) aspect and CTI. The Compound Topographic Index (CTI also called Topographic Wetness Index) is a steady-state wetness index. It involves the upslope contributing area, a slope raster, and a couple of geometric functions. The value of for each cell in the output raster (the CTI raster) is the value in a flow accumulation raster for the corresponding DEM.

21 classes of soil types (level 1) from WRB (FAO, 1998) soil classification system were also used as auxiliary information. These classes are: 1. AB (Albeluvisol), 2. AC (Acrisol), 3. AN (Andosol), 4. AR(Arenosol), 5. CH (Chernozem), 6. CL (Calcisol), 7. CM (Cambisol), 8. FL (Fluvisol), 9. GL (Gleysol), 10. GY (Gypsisol), 11. HS (Histosol), 12. LP (Leptosol), 13. LV (Luvisol), 14. PH (Phaeozem), 15. PL (Planosol), 16. PZ (Podzol), 17. RG (Regosol), 18. SC (Solochack), 19. SN (Solonetz), 20. UM (Umbrisol), 21. VR (Vertisol)). All of the classes were included in the model as they are; they were not reclassified.

10 geological classes of 1/5.000.000 scale International Geological Map of Europe and Adjacent Areas (IGME 5000) were also used as auxiliary information. These classes are: 1. (Meta-) Sedimentary rocks, 2. Acid magmatic and metamorphic rocks, 3. Limestones, 4. Acid to intermediate, 5. Other rocks, 6. Basic magmatic and metamorphic rocks, 7. Ultra-basic magmatic and metamorphic rocks, 8. Intermediate to basic igneous and metamorphic rocks, 9. Intermediate magmatic and metamorphic rocks, 10. Basic to ultra-basic)

The land cover data collected within the CORINE Land Cover (CLC) were also used as auxiliary information. Existing 44 CLC classes were grouped and reclassified into 7 new classes as listed: 1. Urbanized (Corine classes 1–11), 2. Agricultural (Corine classes 12–22), 3. Forest/Natural areas (Corine classes 23–29), 4. Arenosol (Sand) (Corine class 30), 5. Bare rocks/open spaces/glaciers (Corine classes 31–34), 6. Wetlands (Histosols) (Corine classes 35–39), 7. Water bodies (Corine classes 40–44)

Texture information of the soils was also obtained from ESDB. All of the textural classes were used as they appeared in the database. Those 7 classes were; 1. Coarse (18% < clay and > 65% sand), 2. Medium (18% < clay < 35% and > = 15% sand, or 18% <\clay and 15% < sand < 65%), 3. Medium fine (< 35% clay and < 15% sand), 4. Fine (35% < clay < 60%), 5. Very fine (clay > 60%), 6. No mineral texture (Peat soils).

Dominant parent material level 3 classes of ESDB were also included in the study. These 8 classes were; 1. Consolidated-clastic-sedimentary rocks, 2. Sedimentary rocks (chemically precipitated,\evaporated, or organogenic or biogenic in origin), 3. Igneous rocks, 4. Metamorphic rocks, 5. Unconsolidated deposits (alluvium, weathering\residuum and slope deposits), 6. Unconsolidated glacial deposits/glacial drift, 7. Eolian deposits, 8. Organic materials

Precipitation and temperature datasets were derived from two different sources as indicated in the list in previous section as different meteorological records from several temporal intervals and from different resolutions. WorldClim dataset was obtained from EFSA spatial dataset which was made available in May 2011 and created on the basis of the dataset provided by JRC [31]. The whole meteorological dataset contains 27 layers such as Mean monthly temperature (12 maps, each per month), Mean annual temperature, Arrhenius weighted mean annual temperature, Mean monthly precipitation (12 maps, each per month), Mean annual precipitation. The dataset was described in Hijmans et al.[30]. The other dataset was obtained from “The Crop Growth Monitoring System (CGMS)” which is the core of the MARS Crop Yield Forecast System (MCYFS) currently used in forecasting activities in Europe by AGRI4CAST action of JRC. One of the main output of the CGMS system are the Meteorological Interpolated data. The CGMS database contains daily meteorological interpolated data from 1975 to the last calendar year completed, covering the EU Member States, neighboring European countries, and the Mediterranean countries. Several available meteorological parameters (min-max temperature, mean daily vapor pressure, mean daily rainfall, etc.) interpolated to a 25x25 km grids and can be downloaded in the ASCII comma delimited text format.

### Method

The Regression-Kriging method was applied to build the model and assess the distribution of SOC in this study. Supposing that a data vector describing a soil property is a random variable Z, determined at locations in a region, X = x1, …, xN, and consisting of three components as;

$$Z\left(x\right)=m+Z_{1}\;\left(x\right)+\;\varepsilon \;\left(x\right)$$ (1)

where m is the local mean for the region, Z1 (x) is the spatially dependent component and ε the residual error term, spatially independent.

The assumption in the RK technique is that the deterministic component of the target (soil) variable is accounted for by the regression model, while the model residuals represent the spatially varying but dependent component (Z1 in Eq 1). If the exogenous variables used in the regression equation are available at denser locations than the target variable, the equation can then be used to predict the *m* factor of those locations [6].

Multiple linear Regression-Kriging geostatistical technique was used to estimate regression coefficients, calculate residuals, and determine significant predictors for soil organic carbon contents. Regression coefficients are estimations to predict target variable or to explain the variability and spatial correlation in target variable.

RK method was applied to assess and map organic carbon distribution by using 3 different combinations of datasets in EU scale. These combinations were;

1. LUCAS samples,
2. Aggregated samples from LUCAS and CZOs,
3. Aggregated samples from LUCAS, CZOs and BioSoil.

All of the analyses were performed in R 3.1.1 open source software by using several packages such as gstat, mapproj, maptools, rgdal, sp, zoo, xts, space-time, mass. For mapping purposes ArcGIS 10.2.2 (ESRI) software was also used. “Akaike information criterion (AIC)” was used to obtain the best fit for the model in R.

The “repeated random sub-sampling validation” model was used for validating the model, by writing a code in R which takes off 25% validation datasets randomly from the whole dataset and calculate the results for subsets and put the subsets back and again takes off new subsets for 10 times. Final validation result was calculated by taking the averages of the results comes from 25% validation datasets.

## Results

**Fig 3:**
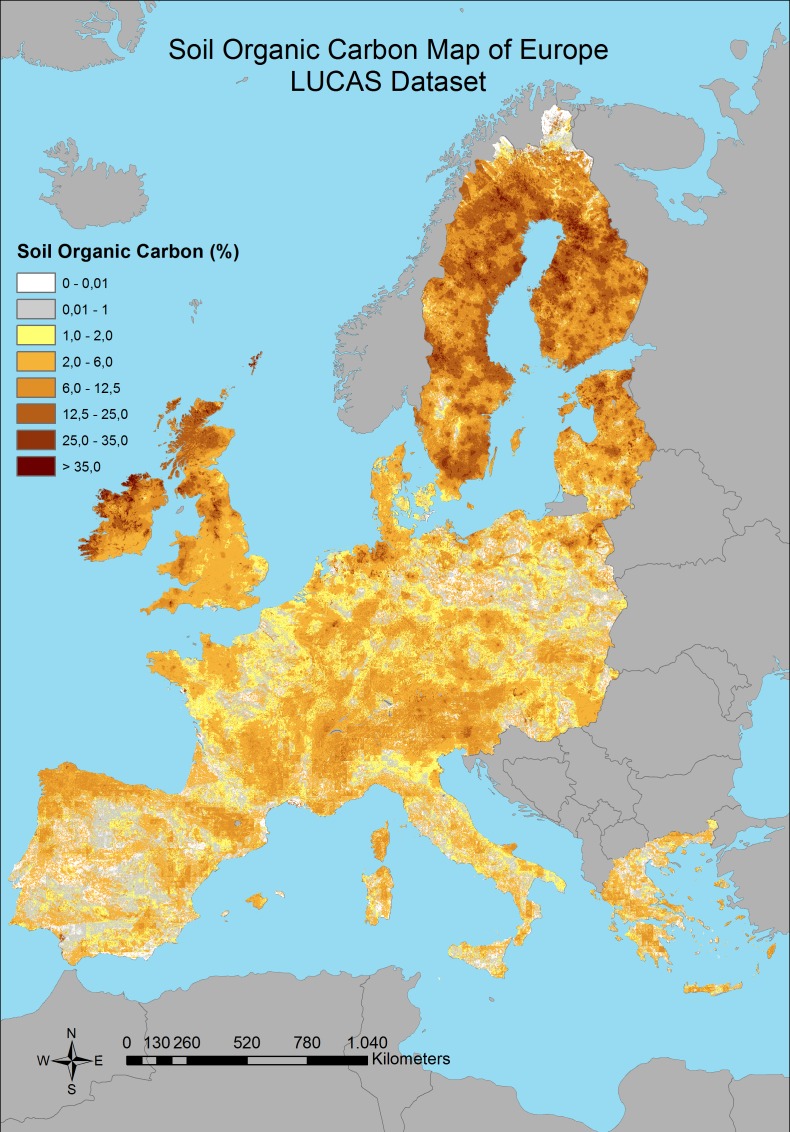
Predicted distribution of SOC content by using combination 1 dataset.

**Fig 4:**
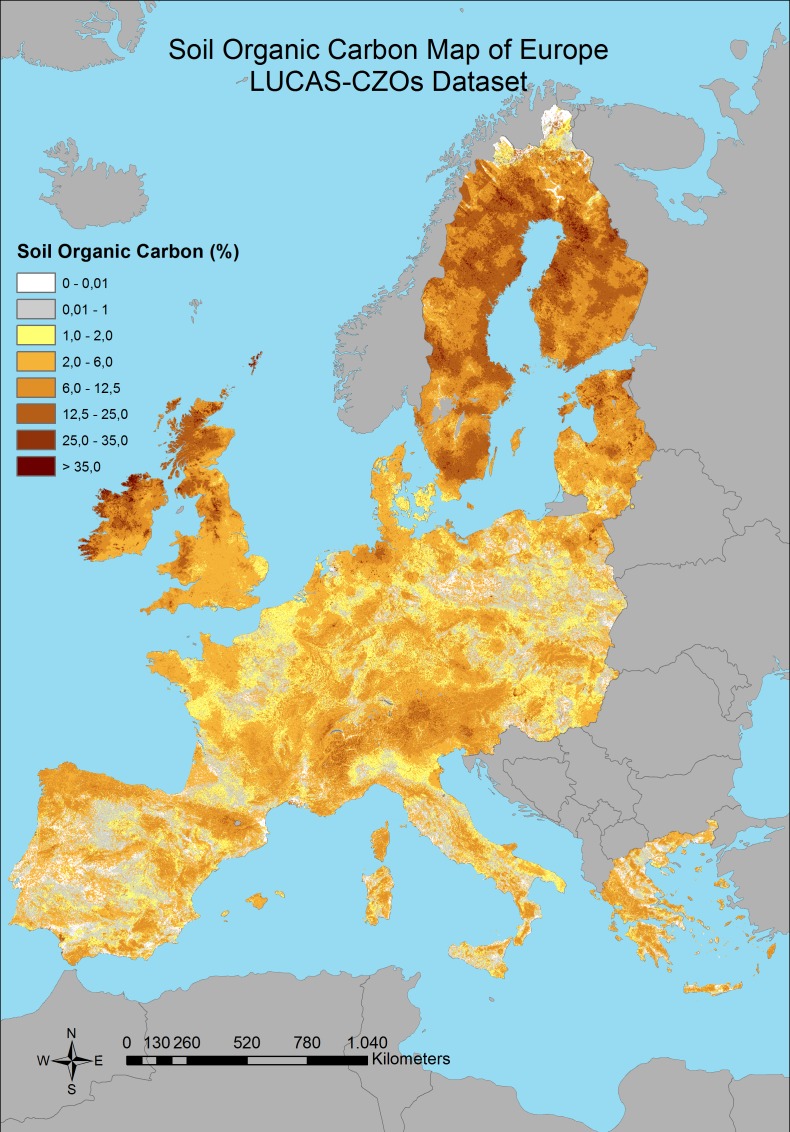
Predicted distribution of SOC content by using combination 2 dataset.

**Fig 5:**
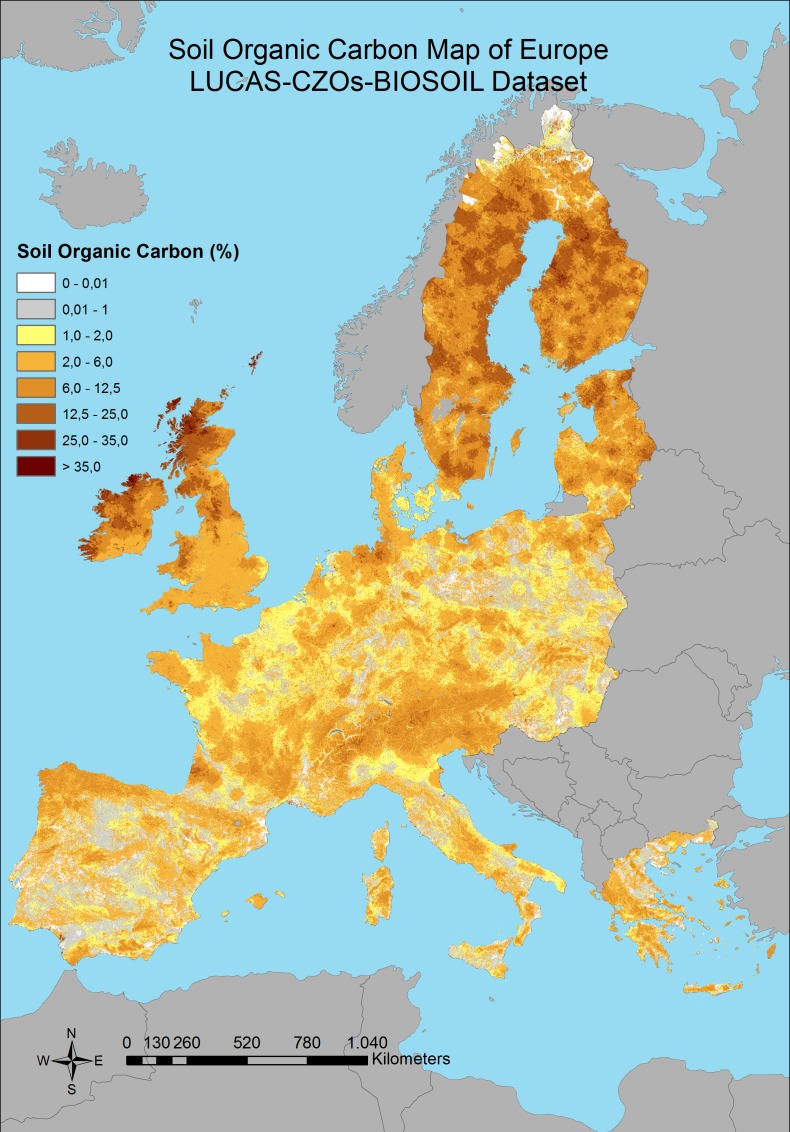
Predicted distribution of SOC content by using combination 3 dataset.

Significant correlation between the covariates and the SOC was found for all of the predictions with different combination of the dataset; with an R^2^, 0.4, 0.41 and 0.33 respectively (p < 0.05) (Table 1), which are better results than found by Brogniez et al. [33] (R^2^ = 0.28). The averages for each of the SOC calculations and standard deviation were founded as 5.73% and 6.08%; 6.08% and 6.02%; 5.46% and 5.34% respectively (Table 1). The final maps which show the SOC distributions with the RK prediction models for three different combinations of dataset can be seen in Figs 3–5.

**Table 1:** Comparison of the results of the predicted maps.

MAPS	R^2^	Standard error	Mean of SOC	Standard deviation of SOC
**LUCAS**	0.4003	0.790	5.73	6.08
**LUCAS+CZOs**	0.4062	0.792	6.08	6.02
**LUCAS+CZOs+BIOSOIL**	0.3326	0.84	5.46	5.34

Statistically significant predictors of the SOC distribution were found for each of the datasets. For the combination 2; elevation, slope, CTI, Average temperature, average precipitation, total precipitation, Texture class 6 (Peat soils), WRB classes 21,10,6 (Vertisol, Gypsisol, Calcisol), CORINE Classes 1,2,4,5,6 predictors were found as statistically significant (p < 0.01) and 41% of the SOC distribution was best explained by these covariates. Aspect, parent material, geological formations, most of the WRB classes and min-max temperature were not recorded as having significant relationship between SOC. The following regression equation was used to predict organic carbon distribution by using the combination 2:

SOC=[8.29−(0.376nELEVATION)−(0.416nSLOPE)+(0.426nCTI)−(3.11nTAVGE)+(1.97nPRECTOTE)−(0.547nTOTPREC)+(4.26TEX6)+(1.89WRB21)+(2.62WRB10)+(2.55WRB06)−(4.37COR1)−(4.63COR2)−(14.0COR4)−(5.94COR5)+(16.6COR6)] (2)

where nElevation is the normalized elevation; nSLOPE is the normalized slope values; nCTI is the normalized CTI values; nTAVGE is the normalized average temperatures for the 1960–1990 years; nPRECTOTE is the normalized average monthly precipitation for the 1960–1990 years; nTOTPREC is the normalized total precipitation for the 1950–2000 years; TEX6 is the texture class corresponds to peat soils; WRB21-WRB10-WRB06 are the WRB classes respectively correspond to Vertisol, Gypsisol, Calcisol; COR1-2-4-5-6 is the CORINE classes respectively correspond to urbanized, Agricultural, Arenosol (Sand), Bare rocks/open spaces/glaciers, Wetlands (Histosols).

Statistically significant predictors of the SOC distribution were found for the combination 3 as; elevation, slope, CTI, Average temperature, average precipitation, total precipitation, Texture class 6 (Peat soils), WRB classes 10,6 (Gypsisol, Calcisol), CORINE Classes 3,4,6 predictors were found as statistically significant (p < 0.01) and 33% of the SOC distribution was best explained by these covariates. Aspect, parent material, geological formations, most of the WRB classes and min-max temperature were not recorded as having significant relationship between SOC.The following regression equation was used to predict organic carbon distribution by using the combination 3:

SOC=[3.76−(0.645nELEVATION)−(0.283nSLOPE)+(0.342nCTI)−(2.93nTAVGE)+(1.84nPRECTOTE)−(0.283nTOTPREC)+(2.73TEX6)+(2.56WRB10)+(2.56WRB06)+(2.68COR3)−(8.73COR4)+(15.22COR6)] (3)

where nElevation is the normalized elevation; nSLOPE is the normalized slope values; nCTI is the normalized CTI values; nTAVGE is the normalized average temperatures for the 1960–1990 years; nPRECTOTE is the normalized average monthly precipitation for the 1960–1990 years; nTOTPREC is the normalized total precipitation for the 1950–2000 years; TEX6 is the texture class corresponds to peat soils; WRB10-WRB06 are the WRB classes respectively correspond to Gypsisol, Calcisol; COR3-4-5-6 is the CORINE classes respectively correspond to Forest, Arenosol (Sand), Wetlands (Histosols).

The residuals derived from the regression analysis were interpolated by kriging using a semivariogram model with -0.0035 average error and 7.84 root mean squared error (RMSE) for the combination 2 (aggregated samples from LUCAS and CZOs). Measured organic carbon content ranged from 0.07% to 58.68% and an average value of the samples was 5.21%, standard deviation 9.45 for combination 2. Besides, estimated results for combination 3 (aggregated samples from LUCAS, CZOs and BioSoil) were found as between 0 and 61.02% and average organic carbon content of Europe has been found as 5.46% which is medium organic carbon content, and standard deviation 5.34 (Table 1).

The highest average SOC contents was found in the wetland areas in three of the maps (Table 2); then in the scrub/ herbaceous vegetation (natural grasslands, moors, etc.) for combination 2 (LUCAS+CZOs) and combination 3 (LUCAS+CZOs+BIOSOIL) maps, forest for combination 1 (LUCAS) map and in the forest areas for combination 2 (LUCAS+CZOs) and combination 3 (LUCAS+CZOs+BIOSOIL) maps, scrub/ herbaceous vegetation (natural grasslands, moors, etc.) for combination 1 (LUCAS) map. Agricultural areas have much lower soil organic carbon content than forest and semi natural areas.

**Table 2:** SOC averages per land-cover for each method.

	AREA (%)	MEAN		
		LUCAS	LUCAS+CZOs	LUCAS+CZOs+BIOSOIL
**Arable Land**	24.9	2.0	2.1	1.98
**Permanent crops**	2.3	1.9	2.0	1.80
**Pastures**	8.5	5.2	5.3	5.28
**Heterogeneous agricultural areas**	10.9	3.1	3.2	3.03
**Forests**	31.1	7.5	7.7	6.75
**Scrub/herbaceous vegetation (natural grasslands, moors, etc.)**	12.3	7.2	8.0	7.49
**Open spaces with little or no vegetation**	1.7	4.4	7.0	6.37
**Wetlands**	2.0	13.5	13.5	10.48

According to our results, Ireland, Sweden and Finland has the highest SOC and Portugal, Poland, Hungary, Spain, Italy have the lowest values with the average 3% (Table 3). Northern Countries with high precipitation and low temperature averages seem that having higher organic carbon amount than warmer southern Countries.

**Table 3:** Comparisons of the SOC averages for each method in terms of NUTS-Level 0.

NUTS0	MEAN		
	LUCAS	LUCAS+CZOs	LUCAS+CZOs+BIOSOIL
**AT- Austria**	5.46	6.53	5.85
**BE-Belgium**	3.66	3.58	3.28
**CH-Switzerland**	5.38	6.29	6.15
**CZ-Czech Republic**	3.49	3.58	3.37
**DE-Germany**	3.83	3.97	3.50
**DK-Denmark**	3.44	3.61	3.31
**EE-Estonia**	10.89	11.25	9.91
**EL-Greece**	3.11	3.94	3.40
**ES-Spain**	3.04	3.51	3.00
**FI-Finland**	11.63	11.99	9.79
**FR-France**	3.46	3.66	3.38
**HU-Hungary**	3.04	2.98	2.21
**IE-Ireland**	13.29	13.51	13.29
**IT-Italy**	3.04	3.74	3.37
**LT-Lithuania**	4.76	5.00	5.91
**LU-Luxemburg**	3.22	3.43	3.01
**LV-Latvia**	7.46	7.65	6.92
**NL-Netherlands**	3.24	3.57	3.30
**PL-Poland**	3.01	3.14	2.91
**PT-Portugal**	2.78	2.91	2.68
**SE-Sweden**	12.52	13.13	11.15
**SI-Slovenia**	5.90	5.97	5.92
**SK-Slovakia**	3.55	3.81	3.22
**UK-United Kingdom**	8.08	8.40	9.59

Predicted data were evaluated with repeated random sub-sampling validation datasets and average R^2^ and RMSE were found as 0.584 and 0.897 for the predictions with only LUCAS samples; 0.486 and 0.76 for the predictions with LUCAS-CZOs samples; 0.401 and 0.578 for the predictions with LUCAS-CZOs-BIOSOIL samples (Table 4) respectively.

**Table 4:** Results of the validation datasets.

MAPS	R^2^	Standard error
**LUCAS**	0.584	0.897
**LUCAS+CZOs**	0.486	0.76
**LUCAS+CZOs+BIOSOIL**	0.401	0.578

## Conclusion

This study showed that the SOC distribution of Europe was successfully mapped using Regression-Kriging method with good accuracy (R^2^ = 0.4, 0.41 and 0.33 (Table 1)). Moreover, these results gave better estimations than the results found by Brogniez et al [33] (R^2^ = 0.28) which is the latest study predicts topsoil organic carbon content of Europe by LUCAS dataset. According to our results for Europe scale, SOC variation is affected by different variables such as elevation, slope, CTI, Average temperature, average precipitation, total precipitation, Texture, WRB and CORINE variables. The models were determined by those variables which played a dominant role in the predictions. SOC amounts were positively correlated to CTI, average precipitation, texture class indicates peat soils, WRB classes and CORINE class indicates Histosols; negatively correlated to elevation, slope, average temperature, total precipitation, and urbanized-agricultural-sand-bare rocks areas in CORINE Land cover for the combination 3.

LUCAS dataset mostly was based on the samples that were taken from agricultural areas. Due to this, the combination of local dataset (CZOs), which includes samples taken from different land-uses (Forest in Lysina, agricultural land in Fuchsenbigl, degraded land in Koiliars and mountain areas in Damma) and LUCAS samples was the good advantage for calibrating the land-use based soil data. The integration of local soil data (at CZO level) improved the SOC estimates in terms of R^2^ evaluation.

The combination of LUCAS (samples mainly taken from agricultural areas) and BioSoil (samples mainly taken from forest) datasets resulted in a combined dataset that could permit our model to perform better at Europe scale in terms of R^2^ evaluation. It was expected that the merged dataset (Biosoil, LUCAS, CZO) would improve the overall results and give a better output since BioSoil covers up the main limitation of LUCAS dataset which has limited samples from forests, but the findings showed the opposite case with the lower R^2^ than the results of only LUCAS dataset. However, the average of the soil organic carbon content of Europe of this combined dataset (Biosoil, LUCAS, CZO) was predicted closer to the measured average which was 5.21 and with lower standard deviation (5.34) (Table 1).

The highest average SOC contents were found in the wetland areas in three of the maps. Agricultural areas have much lower soil organic carbon content than forest and semi natural areas. Ireland, Sweden and Finland has the highest SOC and Portugal, Poland, Hungary, Spain, Italy have the lowest values with the average 3%. Northern Countries with high precipitation and low temperature averages seem that having higher organic carbon amount than warmer southern Countries.

Concluding, even though the predicted models could explain maximum 41% of SOC distribution, RK digital soil mapping technique is still robust and valid method for the big variability of European scale. This study also showed that, increasing the number of the soil samples by using combination of the databases was not always be helpful to increase the statistical significance of the method results for assessing the SOC distribution. However, applying different geostatistical methods for the prediction (cubist model or Partial Least Squares Regression), or selecting different auxiliary variables from different sources might increase the overall results.
